# Measuring use of research evidence in public health policy: a policy content analysis

**DOI:** 10.1186/1471-2458-14-496

**Published:** 2014-05-23

**Authors:** Pauline Zardo, Alex Collie

**Affiliations:** 1 Institute for Safety, Compensation and Recovery Research (ISCRR), Monash University, Level 11, 499 St Kilda Rd, Melbourne, Australia; 2 Department of Epidemiology and Preventive Medicine, Monash University, Level 11, 499 St Kilda Rd, Melbourne, Australia

## Abstract

**Background:**

There are few Australian studies showing how research evidence is used to inform the development of public health policy. International research has shown that compensation for injury rehabilitation can have negative impacts on health outcomes. This study examined transport injury compensation policy in the Australian state of Victoria to: determine type and purpose of reference to information sources; and to identify the extent of reference to academic research evidence in transport related injury rehabilitation compensation policy.

**Methods:**

Quantitative content analysis of injury rehabilitation compensation policies (N = 128) from the Victorian state government transport accident compensation authority.

**Results:**

The most commonly referenced types of information were Internal Policy (median = 6 references per policy), Clinical/Medical (2.5), and Internal Legislation (1). Academic Research Evidence was the least often referenced source of information. The main purpose of reference to information was to support injury treatment and rehabilitation compensation claims decision-making.

**Conclusions:**

Transport injury compensation policy development is complex; with multiple sources of information cited including legislation, internal policy, external policy and clinical/medical evidence. There is limited use of academic research evidence in Victorian state government injury treatment and rehabilitation compensation policies. Decisions regarding compensation for injury treatment and rehabilitation services could benefit from greater use of academic research evidence. This study is one of the first to examine the use of research evidence in existing Australian public health policy decision-making using rigorous quantitative methods. It provides a practical example of how use of research evidence in public health policy can be objectively measured.

## Background

Increasing use of research within public health policy decision-making processes is expected to result in improved public health outcomes [1,2]. Whilst the field of research translation is rapidly developing, to date most of the research examining use of research evidence in public health agencies has been undertaken in the United States and the United Kingdom. At present there are a limited number of studies that have quantified what and how different types of information, including research evidence, are used in Australian policy environments [3-5]. Studies that have been undertaken in Australian public health policy environments have identified a need to build greater capacity for use of research [6-8]. It is argued that context-specific research is needed to inform the design and implementation of interventions aimed at increasing capacity for the use of research [9-11]. Measures of use of research evidence are also critical to assessing the effectiveness of such interventions [12].

Compensable injury is increasingly recognised as a significant public health issue and policy challenge. Approaches to funding or supporting the costs of injury treatment and rehabilitation and loss of income due to injury vary significantly across countries and jurisdictions [13,14]. Countries such as Canada, New Zealand and Australia have ‘no fault’ injury compensation schemes (Personal Injury Education [15]). The health systems in these countries operate quite differently to those that provide comprehensive health treatment and services support, such as the National Health Service in the United Kingdom; where costs relating to injury treatment and rehabilitation and payments for loss of income are primarily covered by the one funding body [14,16,17]. The 2013 Commonwealth Fund Report showed that in Australia ‘out-of-pocket’ health care costs per capita are more than double that in the United Kingdom [16].

Public compensation schemes in Australia provide compensation for the financial and non-financial costs of injury treatment and rehabilitation, including pain and suffering, to people who have been injured in an accident. Compensation is accessed through both compensation claims and common law processes (Personal Injury Education [15]). Australia’s ‘no fault’ public compensation schemes operate alongside Australia’s universal public healthcare (Medicare) scheme and private health insurance, which is optional in Australia [13]. Public compensation policies may overlap with Medicare policies, but also provide compensation for treatment and services not covered by Medicare or private health insurance. A recent study of injured persons undertaken in the Australian state of Victoria showed that 52.5% of the 118 participants accessed the state’s no-fault public compensation scheme to fund their health care costs, 40% were funded via Medicare and 16% via private health insurance [13].

Public compensation schemes develop policies to guide decisions regarding claims for financial compensation for costs related to injury treatment and rehabilitation. Injured persons, clinicians and compensation claims’ decision-makers (claims decision-makers) can utilise compensation policies to determine which injury treatments and rehabilitation services injured persons may be eligible to receive compensation for. It is now recognised that policy regarding the provision of healthcare services to injured people should be informed by research evidence. For example, the Institute for Work and Health in Canada exists to support evidence-informed policy and practice regarding workplace injury and illness prevention and compensation through the production of research, research translation activity and the development of evidence-informed products [18]. In Australia, government healthcare policies, such as those under the Medicare Benefits Schedule which defines the healthcare services available under Australia’s universal Medicare scheme, are now required to be based on research evidence and reviewed within specified timeframes [19].

Examination of the health outcomes for people receiving compensation for injury rehabilitation is a growing area of focus for both researchers and governments. It has been shown that individuals receiving injury treatment and rehabilitation compensation can have poorer health outcomes compared to those who have not received compensation for the same types of injury, including experiences of chronic pain, low back pain, general function etc. [20-23]. Another study found that access to compensation had a protective effect on mortality [24]. These studies cover injury compensation schemes from a range of countries and regions, including the Australian state of Victoria. Two of these studies found that the health outcomes for injured participants receiving compensation remained evident after controlling for the effect of confounding factors such as age, injury severity and injury status [20,24].

Research has shown that a range of complex factors affect the health outcomes of injured persons including: nature and severity of injury; psychosocial factors; employment and/or return to work status; management and education of treatment and rehabilitation; as well as compensation access and experience of compensation processes [23-25]. Other research has shown that macro changes in policy such as legislative amendment can have substantial positive impacts on the health outcomes of injured persons [26,27]. Considering the international push toward evidence-informed policy and practice as a means for achieving improved public health outcomes [28-30]; this research highlights a need to better understand how public compensation policy might support the achievement of improved outcomes for injured persons [23,24].

In Australia, all states and territories have a transport accident compensation system. The estimated economic cost of transport related injury in Australia is $18 billion per year [31,32]. In the state of Victoria approximately 16,000 persons per annum claim compensation from the state transport compensation authority, the Transport Accident Commission (TAC). In 2010/11 the TAC spent $937 million on injury rehabilitation compensation [33]. As such, the TAC plays a critical public health role in Victoria. The types of health care services the TAC funds include: ambulance services, hospital services, medical and pharmacy services, and physiotherapy and other injury rehabilitation therapy and nursing services and individuals with severe injury can receive life-time care [13,34]. Compensation claims for injury treatment and rehabilitation services must meet the eligibility requirements of TAC policies to be approved and paid by the TAC.

Considering the substantial costs of injury compensation; the impact that both policy and compensation can have on health outcomes; and the increasing recognition that public health policies should be evidence-informed where relevant and feasible; there is a pressing need to examine the information that is being used to inform injury treatment and rehabilitation compensation policy development and decision-making. Understanding use of information in compensation policy development and decision-making provides an opportunity to identify how academic research evidence might be used to support future compensation policy development and decision-making and contribute to improved health outcomes for injured persons receiving compensation.

The objectives of this study were to: (1) examine the types of information used in Victorian transport injury treatment and rehabilitation compensation policies; (2) determine the purpose of reference to information in these policies; and (3) determine the level of reference to academic research evidence in these policies. This study forms part of a larger study that has also examined the use of information, including research evidence, in injury prevention and rehabilitation compensation policy and program development through self-reported measures.

## Methods

A quantitative content analysis was undertaken on all TAC injury rehabilitation compensation policy documents existing as at 30 December 2010 (N = 128). TAC policies are designed to outline criteria regarding eligibility for financial compensation for treatment and rehabilitation for persons injured as a result of a transport accident in Victoria. A very broad range of treatment and non-treatment services are covered by the TAC for those who can demonstrate eligibility, ranging from acupuncture and massage to complex surgical treatments and nursing services. See Additional file 1.

Quantitative content analysis seeks to count the occurrence and frequency of specific units of observation, such as words, sentences or phrases, in a unit of analysis, such as policies, newspapers, advertisements and other text based documents [35,36].

### Document selection

TAC injury rehabilitation compensation policy documents formed the unit of analysis. A single copy of each policy was downloaded and saved from the TAC website as at 30 December 2010 [37]. All policy documents collected were printed and coded by hand. Each hyper-link within the policies was checked to ensure its information type and purpose was recorded accurately.

### Categorisation and coding

In content analysis the decision about what to code is driven by the research questions; ‘content analysis is an approach to the analysis of documents and texts that seeks to quantify content in terms of pre-determined categories and in a systematic and replicable manner’ [35]. Based on the research questions and initial coding and review of the first 40 policies collected, the following categories of interest were established: ‘type of information’, ‘purpose of reference to information’ and ‘type of policy’.

Words, sentences and phrases were defined as the unit of observation. Codes were applied to explicit references to words that described ‘type of information’, ‘purpose of reference to information’ and ‘type of policy’. Explicit reference was defined as stated, not implied [36]. Codes were expanded and changed as a result of the initial coding process to ensure codes were ‘exhaustive, exclusive and enlightening’ [36].

#### Coding and counting process

For each of the 128 policies, the title of the policy, and the existence (yes/no) and frequency of each code were recorded.

•Coding for Type of Policy: First, each policy was coded as either ‘treatment’ or ‘non-treatment’. Treatment was defined as any type of treatment, medicine or surgery applied to an individual to physically or psychologically treat the injury. Non-treatment was defined as provision of services or equipment, not directly related to physical or psychological treatment.

•Coding for Type of Information: Next, words, sentences and phrases referring to a ‘type of information’ were coded to describe the type of information referenced. Eight types of information were identified:

•Academic/Scientific Research Evidence: references to published, peer reviewed academic or scientific research evidence or actual citations of published, peer reviewed academic or scientific research evidence.

•Internal Legislation: references to the Transport Accident Act 1986, or references to specific sections of the Act.

•External Legislation: references to acts or regulations other than the Transport Accident Act 1986.

•Internal Policy: references to TAC policies.

•External Policy: references to policies from government agencies (excluding the TAC) and references to policies from professional organisations.

•Clinical/Medical Evidence: references to medical certificates, medical advice, any reference to evidence or information justifying the need for treatment or non-treatment services provided by any medical/clinical practitioner.

•Costs Evidence: references to receipts and other physical evidence of money spent on treatments or non-treatment services.

•Other Evidence: references to evidence or information that were not clearly defined, for example ‘objective evidence’.

•Coding for Information Purpose: Finally, each ‘type of information’ code was then coded for information purpose. That is, each reference to information was identified as either ‘supporting policy development’ or ‘supporting claims decision-making’. The code ‘supporting policy development’ meant the purpose for reference to information was to validate the need for the policy. In contrast, the code ‘supporting claims decision-making’ meant the purpose for reference to information was to support compensation payment decisions made by TAC claim managers. Words and sentences surrounding coded references to information that described the purpose were used to identify ‘information purpose’.

During the initial coding process, it became evident that the policy document structure was largely consistent. Policies included sections entitled ‘Policy’ and ‘Guidelines’. Codes in the ‘Policy’ section were almost always coded as ‘supporting policy’ while codes in the ‘Guidelines’ section were almost always coded as ‘supporting claims decision-making’. To ensure consistency of coding, the primary coder (lead author) determined that all references to information that came under the heading ‘Policy’ and/or prior to the heading ‘Guidelines’ would be coded as ‘supporting policy’ and that references to information under the heading ‘Guidelines’ would be coded as ‘supporting claims decision-making’. Where these headings were absent, the words surrounding a reference to information were used to identify and code ‘information purpose’.

### Inter-rater analysis

An inter-rater analysis was undertaken to test the content analysis method. To determine the number of policies that would need to be inter-rated to detect more than a 20% difference in the coding and counting for each variable between two raters, a power analysis with 80% statistical power at the 5% significance level was performed. This showed 39 or more policies needed to be inter-rated. A second rater independently coded a random sample of 45 policies following the coding system developed by Rater 1 (Lead author).

The difference in coding for type of information and information purpose between Rater 1 and Rater 2 was tested using independent samples t-tests for frequencies and the Mann–Whitney U-Test for categories, set at the 0.05 significance level. There were no significant differences between Rater 1 and Rater 2 for all frequencies and categories, with the exception of the costs category, which was excluded from further analyses.

### Data analysis

Firstly, descriptive analyses were performed to determine the number of references to information per policy and per category of information. Frequency tables and histograms for all counts of ‘type of information’, ‘information purpose’ and ‘type of policy’ were used to analyse frequencies. Cross-tabs were used to analyse categories of ‘type of information’, ‘information purpose’ and ‘type of policy’.

Secondly, a comparison of the type of information referred to in treatment and non-treatment policies was conducted. As the data were positively skewed, the Mann Whitney U Test, set at the 0.05 significance level, was used to test whether there was a statistically significant difference in the median number of references to a type of information in treatment compared to non-treatment policy.

Finally, a comparison of references to a type of information that was identified as ‘supporting policy development’ or ‘supporting claims decision-making’ was conducted. As the data were positively skewed, and the samples related, the Related Samples Wilcoxon Signed Rank Test, set at the 0.05 significance level, was used to test whether there was a statistically significant difference in the median number of references to a type of information in these two categories.

All analyses were performed using Statistical Package for the Social Sciences version 19.0.

## Results

Internal Policy, Clinical/Medical Evidence, Internal Legislation and Other Evidence were the types of information most commonly referenced.

There were 50 references to academic research evidence in 30 policies (23.4%). Academic Research Evidence was the type of information least commonly referenced (Table 1).

**Table 1:** Total references to information in injury treatment and rehabilitation compensation policy

**Information type**	**Frequency (% total) of**	**N (%) policies that**	**Median (range) of reference**
	**reference to information**	**reference information**	**to information per policy**
**Internal policy**	**1133 (47.5)**	**124 (96.9)**	**6 (0–36)**
**Clinical/Medical evidence**	**519 (21.8)**	**108 (84.4)**	**2.5 (0–28)**
**Internal legislation**	**203 (8.5)**	**122 (95.3)**	**1 (0–11)**
**Other evidence**	**175 (7.3)**	**88 (68.7)**	**1 (0–13)**
**External policy**	**245 (10.3)**	**63 (49.2)**	**0 (0–24)**
**External legislation**	**58 (2.4)**	**22 (17.2)**	**0 (0–16)**
**Academic/Scientific research**	**50 (2.1)**	**30 (23.4)**	**0 (0–7)**
**Total**	**2383 (100.0)**	**128 (100.0)**	**15.5 (0–67)**

There were significantly more references to information supporting claims decision- making compared to references to information supporting policy development (p = 0.000).

There were significantly more references to Internal Policy, Clinical/Medical Evidence and Other Evidence supporting claims decision-making compared to references to information supporting policy development.

Internal Legislation and Internal Policy were the types of information most often referenced to support policy development (Table 2).

**Table 2:** References to Information supporting policy development and claims decision-making

**Information type**	**Supporting policy development**			**Supporting claims decision-making**			
	**Frequency (% total) of reference to information**	**N (% total) policies that reference information**	**Median (range) references per policy**	**Frequency (% total) of reference to information**	**N (% total) policies that reference information**	**Median (range) references per policy**	**P Value**
**Internal policy**	**309 (51.5)**	**91 (72.8)**	**1 (0–27)**	**823 (46.2)**	**112 (93.3)**	**4.5 (0–35)**	**p = <0.001**
**Clinical/Medical evidence**	**25 (4.2)**	**19 (15.2)**	**0 (0–3)**	**493 (27.6)**	**103 (85.8)**	**2 (0–28)**	**p < =0.001**
**Internal legislation**	**150 (25.0)**	**115 (92.0)**	**1 (0–11)**	**55 (3.1)**	**32 (26.7)**	**0 (0–4)**	**p < =0.001**
**Other evidence**	**25 (4.2)**	**14 (11.2)**	**0 (0–5)**	**150 (8.4)**	**83 (69.2)**	**1 (0–13)**	**p < =0.001**
**External policy**	**53 (8.8)**	**19 (15.2)**	**0 (0–15)**	**192 (10.8)**	**55 (45.8)**	**0 (0–18)**	**p < =0.001**
**External legislation**	**31 (5.2)**	**13 (10.4)**	**0 (0–16)**	**27 (1.5)**	**14 (11.7)**	**0 (0–5)**	**p = 0.592**
**Academic/Scientific research**	**7 (1.2)**	**4 (3.2)**	**0 (0–3)**	**43 (2.4)**	**29 (24.2)**	**0 (0–5)**	**p = <0.001**
**Total**	**600 (100.0)**	**125 (100.0)**	**3 (0–52)**	**1783 (100.0)**	**120 (100.0)**	**11 (0–65)**	**p = < 0.001**

There were significantly more references to Academic Research Evidence, Clinical/Medical Evidence, External Legislation and Internal Legislation in treatment compared to non-treatment policies (Table 3).

**Table 3:** References to information in non-treatment and treatment policies

**Information type**	**Non-treatment policies**			**Treatment policies**			
	**Frequency (% total) of reference to information**	**N (% total) policies that reference information**	**Median (range) references per policy**	**Frequency (% total) of reference to information**	**N (% total) policies that reference information**	**Median (range) references per policy**	**P Value**
**Internal policy**	**579 (47.7)**	**59 (93.6)**	**6 (0–35)**	**554 (47.4)**	**65 (100.0)**	**6 (1–36)**	**p = 0.785**
**Clinical/Medical evidence**	**214 (17.6)**	**49 (77.8)**	**2 (0–25)**	**305 (26.1)**	**59 (90.8)**	**3 (0–28)**	**p = 0.029**
**Internal legislation**	**115 (9.5)**	**57 (90.5)**	**1 (0–11)**	**88 (7.5)**	**65 (100.0)**	**1 (1–7)**	**p = 0.017**
**Other evidence**	**106 (8.7)**	**43 (68.2)**	**1 (0–13)**	**69 (5.9)**	**45 (69.2)**	**1 (0–3)**	**p = 0.279**
**External policy**	**138 (11.4)**	**28 (44.4)**	**0 (0–24)**	**107 (9.1)**	**35 (53.8)**	**1 (0–17)**	**p = 0.415**
**External legislation**	**52 (4.3)**	**18 (28.6)**	**0 (0–16)**	**6 (0.5)**	**4 (6.15)**	**0 (0–2)**	**p = 0.001**
**Academic/Scientific research**	**10 (0.8)**	**6 (9.5)**	**0 (0–5)**	**40 (3.4)**	**24 (36.9)**	**0 (0–7)**	**p = <0.001**
**Total**	**1214 (100.0)**	**63 (100.0)**	**13 (0–58)**	**1169 (100.0)**	**65 (100.0)**	**16 (3–67)**	**p = 0.852**

## Discussion

### Types of information referenced

Findings showed that Internal Policy and Internal Legislation are heavily relied upon by TAC policy decision-makers when developing transport accident injury rehabilitation compensation policy. It is not surprising that most policies (95.3%) referred to Internal Legislation, as the organisational policies that the TAC develops must comply with the relevant legislation. However, it is interesting that almost all the policies (96.9%) referenced other internal policies, meaning that essentially the organisation was referencing their own prior policy decisions as justification for further policy decisions. Internal Policy was referenced in almost all policies and referenced more frequently than any other type of information. Clinical/Medical Evidence was the second most frequently referenced type of information, more than twice as frequently as Internal Legislation and Other Evidence. Clinical/Medical Evidence was often required by the TAC to verify an individual’s eligibility to claim compensation for a given treatment. To assess clinical/medical evidence provided by medical practitioners, the TAC has developed a Clinical Justification Framework to guide claims managers in decision-making regarding compensation for particular treatment and services. There is also a Clinical Panel comprised of clinical practitioners that advise the TAC on compensation policy development and implementation.

The extent to which Clinical/Medical Evidence, Internal and External Legislation were referenced within treatment policies suggested that development of treatment policies is complex. To develop treatment compensation policies, government policy workers must refer to and comply with the requirements of a raft of other legislation and regulations within the public health system. The complexities of policy development at the health systems level has not been well addressed in the evidence-informed policy literature to date [5,8,9,38-40].

### Purpose of reference to information

The primary purpose of reference to information was to inform decision-making regarding eligibility for compensation for treatment and non-treatment services claimed by injured persons. References to information to support claims decision-making were almost three times as frequent as references to information to support policy development. This indicates that in this context, information is most often used to inform the implementation stage of the policy process [4,5,41]. Clinical/Medical Evidence as well as Internal Policy was used to inform decision-making at this stage, demonstrating that decision-making in public health is inter-dependent. In this context decisions about compensation by claims decision-makers is dependent on the information and evidence provided to them by clinical practitioners.

### Use of academic research evidence

Whilst public health policy development is informed by a range of information types, as well as ideology, politics and community values; internationally there is growing interest in ensuring that wherever possible or relevant, health policy development is informed and/or supported by research evidence [7,28,30,42,43]. In this study it was expected that reference to academic research evidence would be more frequent in treatment-focused policies compared to non-treatment policies. This is because it would be reasonable to expect that policies regarding compensation for transport costs for example, would not need to be informed by research evidence. Whereas specific clinical treatments were expected to be more likely to have available published evidence of effectiveness. Whilst the compensation policies analysed were diverse, categorisation of policies into treatment and non-treatment groups allowed us to compare research use across these two broad types of policies.

Of the 65 treatment policies, 41 (63%) made no reference to Academic Research Evidence. There was also no reference to Academic Research Evidence in 75% of the policies that referenced information to support compensation decision-making. Access to appropriate treatment is critical to effective and efficient recovery from injury. The importance of evidence-informed treatment practice is recognised at the national level in Australian public health policy. The treatment compensation policies existing under the Medicare Benefits Schedule are required to be evidence-based and are subject to regular review to ensure they remain effective and efficient over time, and take account of new evidence [19].

In the thirty policies that referenced academic research, the most common reference was a ‘recent peer reviewed journal article’; this was a generic statement within the policies and not a reference to specific articles or studies. Only six of the 30 policies that referred to academic research cited published articles or specific studies. This demonstrates that the main quality requirement for academic research evidence used to inform decision-making is ‘recent’ and ‘peer reviewed’; only two policies made reference in the policy text to ‘clinical trials’, ‘clinical research’, or ‘high quality evidence’. Whilst it is critical that evidence applied is peer reviewed and of publishable quality, how recently the study was published is not an appropriate quality indicator. The quality of treatment effectiveness evidence is dependent on research methods. Hierarchies of evidence place ‘gold standard’ research methods such as systematic reviews and randomised controlled trials (RCTs) at the top [44].

Whilst RCTs or systematic reviews are not always an appropriate method for answering public health policy questions [5,45,46], high quality evidence of treatment effectiveness is particularly relevant to injury rehabilitation compensation policy. If particular treatments lack high quality research evidence demonstrating their effectiveness, governments can use this information to cease or decline to provide compensation for that treatment. Conversely, governments can use high quality research evidence to identify and provide compensation for treatments that have been shown to be effective. Ensuring that compensation policies are informed by the best available research evidence wherever possible is important for supporting both claims and clinical decision-making, as it has been established that clinicians may not always recommend or provide care that is aligned with current best practice [29,42,47]. There is need for further research on clinical decision-making regarding healthcare for persons receiving compensation.

The limited reference to published academic research suggests there is an opportunity to increase use of research evidence in transport accident injury rehabilitation compensation policy in Victoria. Whilst policy development in this context may have been informed by research evidence not referenced in the policy document, and there would be instances where it is not possible or appropriate to use research evidence, referencing the information used to inform the development of the policy is necessary to ensure decision-making is transparent. Increased use of research evidence in compensation policy development may be facilitated through the development of evidence quality standards and the establishment of regular policy review processes, such as that undertaken by the Australian pharmaceutical and medical benefits schemes [19,48,49] and informed by published literature on hierarchies of evidence [44]. In the state of Victoria there are no externally applied guidelines or standards that must be complied with for the development of operational level government policy such as TAC compensation policies.

The limited reference to academic research evidence in the compensation policies analysed in this study may also be attributed to well-known barriers to use of research evidence in health policy decision-making. Policy workers consistently cite lack of time and lack of skills in assessing research evidence as a barrier to use of evidence [6,7,38,50]. Policy workers often find that research is not relevant to their particular needs and the recommendations of research are not actionable in their context [5,38,50]. Further, whilst there is much existing academic research evidence on public health problems, significant gaps remain [5,51,52].

Whilst policy is but one component of the compensation decision-making process, and that in this context the TAC’s Clinical Justification Framework and Clinical Panel also support claims decision-making; developing systems and processes to ensure that wherever possible compensation policies are informed by the best available evidence can support both policy development and implementation. One of the key reasons for the development of the Clinical Justification Framework was to ensure that injured persons were receiving treatment and management that would be ‘goal oriented, evidence based and clinically justified’ [53]. Research evidence can assist decision-making regarding whether a compensation policy should be developed for a particular treatment or service, and can also support decisions regarding the development of policy content.

Increased reference to and transparency regarding the information types used to inform policy development could enhance consistency and efficiency. Claims decision-makers could refer to the evidence-informed policies to make individual case decisions about whether compensation can be paid for particular treatments and services; rather than having to seek advice from the Clinical Panel or searching research literature to determine if the particular treatment or service met the requirements of the Clinical Justification Framework. It also supports clinicians and injured or ill persons who are eligible for compensation to make informed decisions regarding treatment and rehabilitation services they seek to access.

There is a continued need for support in the application of research evidence in health policy environments [11,50,51]. Government organisations are subject to external pressures and competing interests that often require them to develop a policy response to address public need and concern, despite a lack of research evidence on the issue at hand [5,41,54]. This study provides a practical example of how use of research evidence can be assessed objectively (as opposed to self-report measures) in a public health policy environment. This data also provides a baseline from which change in use of research in this environment can be measured following the implementation of research translation interventions. There remains a need for the development of evidence-based context-specific interventions and tools that can effectively increase and support use of research evidence in public health policy environments [5,38,55,56].

## Conclusion

In the Victorian compensation policy development context there is an opportunity to improve the capacity for use of academic research evidence by policy decision-makers, potentially through the development of evidence quality standards and regular policy review processes. Specifically, government decision-making about what injury treatment and non-treatment services will be compensated for could be better informed by high quality research evidence. Interventions aimed at increasing the use of research evidence in this context must target capacity for assessing the quality of academic research evidence and increase access to and awareness of academic research evidence relevant to injury rehabilitation compensation policy.

Academic research evidence can provide an important counter weight to other information types used to inform injury rehabilitation compensation policy. However, it is important for those seeking to influence use of research evidence in compensation policy development to be cognisant of the fact that policy development in this context is complex and interdependent, and that academic research will only ever be one of several inputs into the policy process. To be actionable by government, academic research findings and recommendations must address the system level, jurisdictional constraints that compensation policy workers must work with in the development and implementation of compensation rehabilitation policy.

Improving the quantity and quality of research evidence used to inform policy development and claims decision-making in this context has the potential to result in improved compensation health outcomes. Evidence of effectiveness can be used to guide injured persons receiving compensation toward treatments and services that can improve their recovery and away from treatments and services that show no evidence of effectiveness. Whilst policy is just one component contributing to the health outcomes experienced by those receiving compensation; it is a foundation for injury rehabilitation compensation practice and decision-making that can influence the behaviour of claims decision-makers, health care providers, lawyers and others involved in the injury treatment and rehabilitation compensation process.

## Competing interests

The authors declare that they have no competing interests.

## Authors’ contributions

PZ led data collection and analysis. AC contributed to interpretation of results. Both authors contributed to conception of the project, writing and review of the manuscript. Both authors read and approved the final manuscript.

## Pre-publication history

The pre-publication history for this paper can be accessed here:

http://www.biomedcentral.com/1471-2458/14/496/prepub

## Supplementary Material

Additional file 1 List of all TAC policies as at 30 December 2010. Click here for file
