# Supplementary material for: Measuring use of research evidence in public health policy: a policy content analysis
Source: BMC Public Health. 2014 May 23;14:496. doi: 10.1186/1471-2458-14-496 (PMC4048040; doi:10.1186/1471-2458-14-496)
Supplement: Additional file 1 — List of all TAC policies as at 30 December 2010. [file 1471-2458-14-496-S1.docx]

**Additional File**

| **Treatment Policies** | **Non-Treatment Policies** |
| --- | --- |
| Acupuncture | Ambulance |
| Autologous Blood Testing for Surgical Procedures | Blood alcohol Content Reading |
| Non-Established, New or Experimental Treatment | Community Health Centres |
| Blood Drainage - Reinfusion | Criteria for Senior Medical Consultants Higher Fee Rate |
| Bone Growth Stimulation | Dose Administration Aids |
| Botulinum Toxin | Equipment (Medical) |
| Breast Implant Replacement Surgery | Equipment (Medical) Hearing Aids |
| Pharmacy (Chemist medications) | Equipment (Medical) - Life Support Equipment |
| Chiropractic | Equipment (Medical) Mobility and Aides |
| Combination of Compensable and Non-Compensable Treatment | Equipment (Medical) - Operation, Maintenance and Repair Cost |
| Concurrent Physical Treatment by Health Care Providers | Equipment (Medical) - Optical Expenses |
| Contraceptive Treatment | Equipment (Medical) - Prosthetic Equipment |
| Dental Services | Equipment (Medical) - Room Temperature Control Equipment |
| Facial Prosthesis and Prosthetic Digits | Hire Charges (Surgical Equipment) |
| Family Counselling | Hospitals |
| Feldenkrais | Hospitals - Private (Contracted) |
| Functional Electronic Stimulation (FES or Neuro- Muscular Electronic Stimulation | Hospitals - Private (Arrangement) |
| Gait Analysis | Hospitals - Private (Non-Arrangement) |
| Hip Replacement Surgery | Hospitals - Public |
| Human Donor Tissue | Interstate Services |
| Hydrotherapy (Aquatic Physiotherapy) | Medical Imaging |
| Hyperbaric oxygen Therapy | Medical Practitioners |
| Implantable Pain Therapy | Medical Reports Non-TAC Requested |
| Implantable Therapy for Neurological Disorders | Medical Reports Requested by the TAC |
| Infusion Therapy for Pain Management | Equipment (Medical) - Optical Expenses |
| Interpreting Services | Physiotherapy Treatment Notification Plan |
| In Vitro Fertilisation | Road Accident Rescue Services (RAR) |
| Joint Fluid Therapy | Time Limit to Apply for the Payment of Medical and Like Expenses |
| Ketorolac | Appendix - Authorised Service Provider Eligibility |
| Laser Resurfacing (CO2 Laser Procedure) | Audiology |
| Massage Myotherapy | Benefit and Support Service Assessments |
| Maxillofacial Services | Equipment (Rehabilitation) - Computer Equipment |
| Medication to Treat Erectile Dysfunction | Dietician |
| Mental Health (Psychology) | Driving Programs |
| Network Psychology | Drug and Alcohol Services |
| Nursing | Education Support Services |
| Optometry | Equipment (Rehabilitation) Policy |
| Oestrogenic Protein Device (also referred to as OP-1 or BMP-7) | Family Support Workers |
| Osteopathy | Home Modifications |
| Physiotherapy | Modifications or Contribution Towards the Purchase Cost of a Vehicle |
| Podiatry | Occupational Therapy Services |
| Pregnancy (Termination of) | Equipment (Rehabilitation) - Therapeutic Equipment |
| Pro-osteon | Rehabilitation camps |
| Radiofrequency Denervation | Return to Work Programs |
| Sedatives (including Hypnotics and Anti-anxiety Medications) | Schedule 1 Authorised Rehabilitation Services |
| Special food and Special Food Formula | Social Work |
| Spinal Injection Therapies | Special Education Consultant Services |
| Surgery Elective | Equipment (Rehabilitation) - Sporting and Recreation Equipment |
| Surgically Implanted Prosthesis | Therapy Support Services |
| Topical Non-Steroidal Anti-Inflammatory Drugs (Gels, Creams) | Equipment (Rehabilitation) - Therapeutic Equipment |
| Transdermal Topical Preparations (Topical Analgesic Creams) | Vocational Retraining and Pre-Vocational Training |
| Treatment of Transport Accident Injuries with Drugs of Dependency | Attendant Care |
| Vaccinations | Attendant Care Overseas |
| Vitamins, Mineral and Complementary Medications | Case Management |
| Weight Loss Treatment and Services Policy | Community Access |
| Exercise Physiology | Daily Living Expenses |
| Exercise Programs - Supervised | Guide Dogs and Assistance Dogs |
| Gym Swimming and Exercise in Rehabilitation programs | Residential Care |
| Network Occupational Therapy Services | Respite Services |
| Network Pain Management Programs and Pain Management Service | Schedule 2 Authorised Disability Services |
| Occupational Physiotherapy | Schedule 3 - Individual Funding Agreements |
| Orientation and Mobility | Self-Purchasing - Individual Funding Agreements |
| Orthoptics | Self-Purchasing - Individual Funding Agreements Brokerage |
| Network Pain Management Programs |  |
| Speech Pathology |  |
